# Comparison of Human Urinary Exosomes Isolated via Ultracentrifugation Alone versus Ultracentrifugation Followed by SEC Column-Purification

**DOI:** 10.3390/jpm12030340

**Published:** 2022-02-24

**Authors:** Kun Huang, Sudha Garimella, Alyssa Clay-Gilmour, Lucia Vojtech, Bridget Armstrong, Madison Bessonny, Alexis Stamatikos

**Affiliations:** 1 Department of Food, Nutrition, and Packaging Sciences, Clemson University, Clemson, SC 29634, USA; kunh@g.clemson.edu; 2 Prisma Health, Pediatric Nephrology, Greenville, SC 29615, USA; sudha.garimella@prismahealth.org; 3 Department of Epidemiology & Biostatistics, Arnold School of Public Health, University of South Carolina, Columbia, SC 29208, USA; claygila@mailbox.sc.edu (A.C.-G.); bessonny@email.sc.edu (M.B.); 4 Department of Obstetrics & Gynecology, University of Washington, Seattle, WA 98109, USA; luciav@uw.edu; 5 Department of Exercise Science, Arnold School of Public Health, University of South Carolina, Columbia, SC 29208, USA; ba12@mailbox.sc.edu

**Keywords:** biological marker, microRNA, renal failure

## Abstract

Chronic kidney disease is a progressive, incurable condition that involves a gradual loss of kidney function. While there are no non-invasive biomarkers available to determine whether individuals are susceptible to developing chronic kidney disease, small RNAs within urinary exosomes have recently emerged as a potential candidate to use for assessing renal function. Ultracentrifugation is the gold standard for urinary exosome isolation. However, extravesicular small RNA contamination can occur when isolating exosomes from biological fluids using ultracentrifugation, which may lead to misidentifying the presence of certain small RNA species in human urinary exosomes. Therefore, we characterized human urinary exosomal preparations isolated by ultracentrifugation alone, or via ultracentrifugation followed by size exclusion chromatography (SEC) column-purification. Using nanoparticle tracking analysis, we identified SEC fractions containing robust amounts of exosome-sized particles, that we further characterized using immunoblotting. When compared to exosomal preparations isolated by ultracentrifugation only, SEC fractionated exosomal preparations showed higher levels of the exosome-positive marker CD81. Moreover, while the exosome-negative marker calnexin was undetectable in SEC fractionated exosomal preparations, we did observe calnexin detection in the exosomal preparations isolated by ultracentrifugation alone, which implies contamination in these preparations. Lastly, we imaged SEC fractionated exosomal preparations using transmission electron microscopy to confirm these preparations contained human urinary exosomes. Our results indicate that combining ultracentrifugation and SEC column-purification exosome isolation strategies is a powerful approach for collecting contaminant-free human urinary exosomes and should be considered when exosomes devoid of contamination are needed for downstream applications.

## 1. Introduction

Chronic kidney disease (CKD) is a permanent, irreversible condition that involves a gradual loss of kidney function [1]. CKD is thought to afflict millions of Americans annually and many individuals are unaware that they have this condition [2]. There are also no feasible treatments for CKD [1,2]. Patients suffering from CKD may be eligible for kidney transplantation, but some patients who have CKD are at risk of succumbing to this condition before a compatible kidney is available for these individuals due to the lengthy wait time for kidney transplants [3]. There are also many patients afflicted with CKD who are deemed ineligible for kidney transplantation [4,5]. Therefore, the predominant treatment for CKD once the kidneys have failed is dialysis [6]. However, dialysis is both burdensome and exhausting on patients, is expensive, and needs to be performed as a regular, life-long therapy [7,8]. Life expectancy and quality-of-life are also drastically decreased in dialysis patients, too [9,10,11].

While there are several risk factors for CKD, many individuals who develop these risk factors do not acquire CKD [12]. Clinical biomarkers are sometimes used to assess whether a patient may later develop CKD, but some of these biomarkers are collected invasively and have not necessarily been shown to be accurately reliable predictors to determine whether a person may eventually develop CKD [13]. However, data have shown that various dietary and lifestyle patterns may delay or even prevent the onset of CKD [14,15,16]. Therefore, identifying a simple, economical, and non-invasive biomarker to accurately predict who is prone to developing CKD may dramatically decrease the number of people diagnosed with this condition. Indeed, if individuals are informed that they are susceptible to developing CKD, then they would be much more likely to make dietary/lifestyle changes to reduce the chance of acquiring this disease.

Emerging evidence has shown that the small RNA within human urinary exosomes are promising candidates for assessing kidney function [17,18,19]. Furthermore, we and others have been successful with detecting microRNA and other small RNA within intact exosomes isolated from various types of biological fluids [20,21,22,23]. Urinary collection is easy, inexpensive, and non-invasive, and so there are many advantages to using human urinary exosomal small RNA in a clinical setting. Therefore, if various urinary exosomal microRNA are later determined to be biomarkers for CKD, as well as other diseases, such as cancer or atherosclerosis, then this may greatly assist nephrologists, oncologists, cardiologists, and other types of physicians with diagnosing certain conditions. There are numerous strategies for isolating exosomes from human urine and other biological fluids, with ultracentrifugation being considered the “gold standard” for exosome isolation [24,25,26,27]. However, while isolating exosomes via ultracentrifugation is relatively uncomplicated and straightforward, exosomes collected by ultracentrifugation may result in exosomal preparations which contain contamination [28]. A more novel technique for exosome isolation is size exclusion chromatography (SEC) [29], but routine columns used for SEC are unable to accommodate the large urinary volumes needed for exosome isolation. Therefore, we decided to combine ultracentrifugation and SEC column-purification approaches to isolate exosomes from human urine and then compare the isolated exosomes to human urinary exosomes isolated by using ultracentrifugation only. Our results show that while both ultracentrifugation alone and ultracentrifugation followed by SEC column-purification results in collecting robust numbers of human urinary exosomes, only SEC column-purified fractions were shown to be devoid of contamination. Based on these results, we conclude that combining ultracentrifugation and SEC column-purification strategies to isolate exosomes from human urine is superior for obtaining contamination-free exosomal preparations when compared to isolating exosomes from human urine using ultracentrifugation only.

## 2. Materials and Methods

### 2.1. Subject Recruitment

This study was approved by the Institutional Review Board at Prisma Health. We recruited children between the ages of 5–18 years who were attending a primary care clinic. Participants were recruited using fliers and advertisements placed in clinic waiting rooms. Participants were reimbursed $50 for completing the assessment. Children were eligible if they had obesity (BMI > 95th %ile) and were presenting for a well child visit. We selected young age and obesity as inclusion criteria, as childhood and adolescent obesity has been implicated to increase the risk of later developing CKD [30,31,32]. Exclusion criteria included a diagnosis of hypertension, diabetes, or known kidney or heart disease. Subjects were instructed to come for two study visits 3 months apart. However, for this respective study, a sub-set of samples initially collected during visit 1, was used for analyses. Participants (*n* = 5) ranged in age from 7 to 15 (mean age = 11; SD = 3.2). Three of the five participants were male. The subjects had an average BMI of 35.1 (SD = 11.5). Four of the five participants self-identified their race as Black or African American, and one subject identified as Hispanic/Latinx. Individual level demographics are presented in Table 1.

### 2.2. Human Urinary Exosome Isolation

Prior to data collection, informed consent was obtained from primary caregivers and children provided verbal assent. Following the consent procedures, participants completed health questionnaires to assess diet and exercise habits. Anthropometric measurements were collected by trained research staff and BMI was calculated as weight (in kilograms) divided by height (in meters) squared. Participants were mailed sterile urine cups ahead of their visit. They received instructions on how to collect a first morning void and bring it to the visit. Once urine was collected from the human subjects, we immediately transported the urine to Clemson University at 4 °C, so that the urine could be used for human urinary exosome isolation. Briefly, we spun 50 mL of each participant’s voided urine via serial centrifugation at 4 °C, using the following times and speeds per respective spin: (1) 1000× *g* for 10 min; (2) 17,000× *g* for 15 min; (3) 200,000× *g* for 60 min; (4) 200,000× *g* for 60 min. For the first two spins, any pelleted material was discarded, and urine was collected for subsequent spins. For the third spin, exosomes were pelleted, an aliquot of exosome-depleted human urine was collected to use for downstream analysis, and the remaining urine was discarded. The fourth spin consisted of washing the pelleted human urinary exosomes with PBS during this final spin, collecting an aliquot of the PBS wash to use for downstream analysis, removing the remaining PBS wash, and resuspending the washed human urinary exosomes with fresh PBS. Once resuspended, half of the volume of the human urinary exosomal preparations were stored at −80 °C to later use for downstream analysis, while the other sample halves were SEC column-purified using Exo-spin™ mini-HD columns (Cell Guidance Systems LLC, St. Louis, MO, USA) and following manufacturer instructions. Since the manufacturer instructions state fractions 6 and 7 are predicted to have the largest amounts of purified exosomes, we collected both of these fractions, as well as the fractions 5 and 8 because they flanked fractions 6 and 7 and stored all these fractions at −80 °C, so that they may later be used for downstream analysis.

### 2.3. Assessing Human Urinary Particle Size and Number

Human urinary particle number and diameter size were assessed as previously described [22]. Briefly, human urinary exosomal preparations, exosome-depleted human urine (by ultracentrifugation), and PBS used to wash exosomal preparations during ultracentrifugation were analyzed via nanoparticle tracking analysis (NanoSight NS300 instrument; Malvern Instruments, Malvern, UK) [33]. Human urinary exosomal preparations were initially vortexed and serially diluted with molecular biology grade water and the diluent acted as a negative control. Measurements were taken in replicate fashion, with mean values being calculated.

### 2.4. Immunoblotting

Protein concentrations within human urinary exosomal preparations were measured with a BCA assay kit (BioVision, Milpitas, CA, USA). We separated equal amounts of human urinary exosomal proteins using SDS-PAGE as previously described [34]. These separated proteins were subsequently transferred onto PVDF membranes (Merck Millipore Ltd., Burlington, MA, USA) that were incubated in blocking buffer [34] before being probed with the primary antibody mouse anti-CD81 (1:500 dilution, sc-166029; Santa Cruz Biotechnology, Dallas, TX, USA) and primary antibody mouse anti-calnexin (1:1000 dilution, sc-46669; Santa Cruz Biotechnology). We subsequently washed the membranes with TBST after finishing the primary antibody incubation steps and then incubated the membranes with an HRP-conjugated goat anti-mouse IgG secondary antibody (1:5000 dilution, AP181P; Sigma-Aldrich, St. Louis, MO, USA). After the secondary incubation step was complete, we washed membranes with TBST and then incubated the membranes in ECL (Immobilon ECL UltraWestern HRP Substrate; MilliporeSigma, Billerica, MA, USA). We used a ChemiDoc (Analytik Jena US, Upland, CA, USA) for imaging membranes post-ECL incubation [34].

### 2.5. Analyzing Human Urinary Exosome Appearance

To confirm the identity of exosomes within fractionated human urinary exosomal preparations, we imaged the particles in these preparations using transmission electron microscopy [35] and performed this analysis as previously described [22]. Briefly, we pooled together all six and seven human urinary exosomal fractions and added 4% PFA to this pooled sample. We then deposited the particles mixed with 4% PFA by airfuge on Formvar-carbon coated transmission electron microscopy grids (Ted Pella, Redding, CA, USA). We then subsequently embedded and contrasted using a uranyl-oxalate solution (pH 7; Electron Microscopy Sciences, Hatfield, PA) for 5 min, and then subsequently performed another treatment using methyl-cellulose-uranyl-acetate (Sigma-Aldrich) on ice for 10 min. Micrograph images were acquired with a JEM-1400 transmission electron microscope (JEOL, Tokyo, Japan) at 120 kV and an Ultrascan 1000XP digital camera (Gatan, Pleasanton, CA, USA).

## 3. Results

### 3.1. Particle Size Comparisons of Human Urinary Exosomal Preparations Isolated via Ultracentrifugation That Remain Unpurified versus SEC Column-Purified

**Figure 1:**
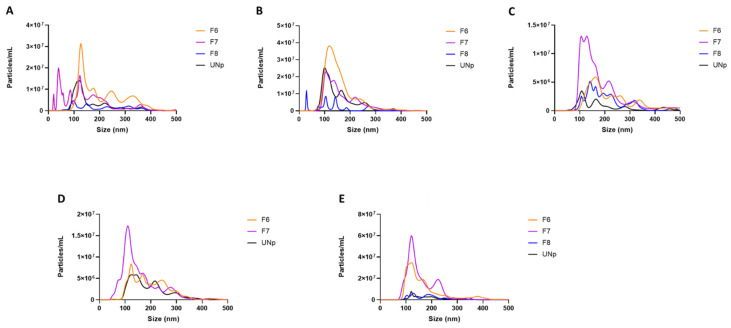
Size distribution of human urinary particles isolated by ultracentrifugation alone versus ultracentrifugation followed by SEC column-purification. (**A**–**E**) Human urine was collected from subjects listed in Table 1. Particle diameter size and concentration determined by nanoparticle tracking analysis for human urinary particles isolated using ultracentrifugation and remained unpurified (UNp) or isolated via ultracentrifugation and then fractionated using SEC column-purification, with fraction 6 (F6), fraction 7 (F7), and fraction 8 (F8) being analyzed. A 500 nm size cutoff was used for data representation. (**D**) F8 was below the limit of detection.

We used nanoparticle tracking analysis by NanoSight to determine whether the human urinary exosomal preparations we collected using either ultracentrifugation alone or SEC column-purification post-ultracentrifugation contained large numbers of exosome-sized particles. Interestingly, fraction 5 of the SEC column-purified human urinary exosomes was under the limit of detection for NanoSight to accurately measure particle number and diameter size within this fraction. However, fractions 6 and 7 demonstrated a similar diameter size pattern and overall particle number profile when compared to human urinary exosomal preparations isolated via ultracentrifugation only, while we observed a relatively inferior particle number and diameter size in fraction 8 when this fraction was compared to all the other preparations (Figure 1A–E and Supplementary Figure S1A–E). When we performed nanoparticle tracking analysis on exosome-depleted human urine (by ultracentrifugation) and PBS used to wash exosomal preparations during ultracentrifugation and then compared the particle numbers of these control samples to the average particle number of the human urinary exosomes isolated using ultracentrifugation/SEC, there were >95% fewer particles detected within the control samples (data not shown). These results imply that SEC column-purification can successfully fractionate human urinary exosomes isolated via ultracentrifugation, resulting in certain fractions being collected that contain robust numbers of exosome-sized particles.

### 3.2. Human Urinary Exosomal Preparations Isolated Using Ultracentrifugation Followed-by SEC Column-Purification Are Devoid of Contamination

**Figure 2:**
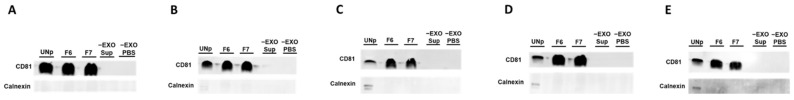
Human urinary exosomal preparations characterized by immunoblotting. (**A**–**E**) Human urine was collected from subjects listed in Table 1. Immunoblotting of lysates of human urinary particles isolated using ultracentrifugation and remaining unpurified (UNp) or isolated via ultracentrifugation and then fraction 6 (F6) and fraction 7 (F7) collected by SEC column-purification. Immunoblotting controls were exosome-depleted human urine (–EXO Sup) and PBS used to wash exosomal preparations (–EXO PBS). Blots were probed for the exosome-positive marker CD81 and exosome-negative marker calnexin.

To assess whether our human urinary exosomal preparations are potentially contaminated with non-exosomal proteins, we used immunoblotting to probe for the exosome-negative marker calnexin, which has been identified in human urinary microvesicle/extracellular vesicle preparations [36]. We also used immunoblotting to probe for the exosome-positive marker and tetraspanin CD81, which has been shown to be present in human urinary exosomes [37]. For immunoblot analyses, we used the human urinary exosomal preparations which had the highest numbers of exosome-sized particles based on nanoparticle tracking analysis, in addition to exosome-depleted human urine (by ultracentrifugation) and PBS used to wash exosomal preparations during ultracentrifugation, which acted as controls. For our results, we detected higher amounts of CD81 protein in fractions 6 and 7 when compared to the human urinary exosomal preparations isolated by ultracentrifugation only, but no protein was detected in the control lanes. However, when we probed for calnexin, this marker was not detected in fractions 6 and 7 or control samples but was detected in some of the human urinary exosomal preparations isolated by ultracentrifugation alone, which suggests contamination is present within the human urinary exosomal preparations that were isolated via ultracentrifugation only (Figure 2A–E).

### 3.3. Assessing the Presence of Exosomes in Pooled SEC Fractionated Preparations by Transmission Electron Microscopy

**Figure 3:**
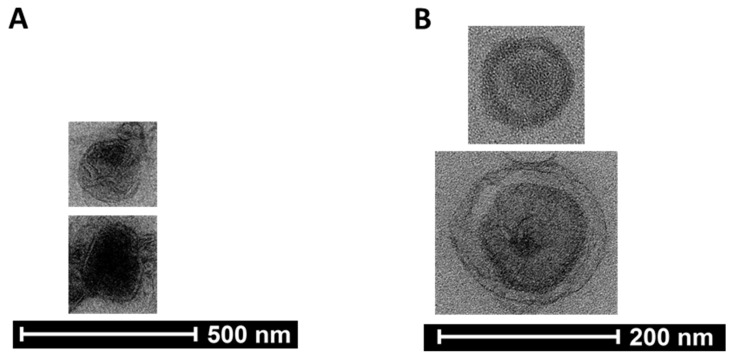
Pooled human urinary exosomal preparation imaged using transmission electron microscopy. (**A**,**B**) Human urinary particles were isolated by ultracentrifugation and further purified using SEC columns. Fraction 6 and fraction 7 were pooled among all collected samples and the pooled preparation was imaged via transmission electron microscopy. The transmission electron micrographs shown are cropped images.

To confirm the presence of exosomes within preparations collected by SEC column-purification post-ultracentrifugation, we pooled all fractions 6 and 7 previously used for nanoparticle tracking analysis and immunoblotting to image via transmission electron microscopy. Our imaging analysis identified exosome-sized particles that exhibited distinct exosome characteristics [22] (Figure 3A,B and Supplementary Figure S2A–D), which indicates human urinary exosomes are present in the fractionated preparations that were assessed by transmission electron microscopy.

## 4. Discussion

In this study, we wanted to test whether human urinary exosomes which were collected via SEC column-purification post-ultracentrifugation provide preparations that are comparable to human urinary exosomes which were isolated using ultracentrifugation only. In our experiments, we observed fractions 6 and 7 having robust numbers of exosome-sized particles that were similar to the numbers of exosome-sized particles detected in human urinary exosomal preparations isolated by ultracentrifugation alone. However, when we assessed protein levels of the exosome-positive marker CD81, we detected higher amounts of this protein in fractions 6 and 7 when compared to preparations isolated by ultracentrifugation only. Moreover, while we did not detect the exosome-negative marker calnexin in fractions 6 and 7, we did observe this protein being present in some of the preparations which were isolated by ultracentrifugation alone, which implies contamination. We also used transmission electron microscopy to image particles within fractions 6 and 7, which confirmed exosomes were present in these preparations. Therefore, we conclude that isolating human urinary exosomes via ultracentrifugation followed by SEC column-purification is superior to ultracentrifugation alone when attempting to collect human urinary exosomal preparations that are free of contamination.

While conventional ultracentrifugation is considered the gold standard for isolating exosomes from biological fluids [24,25,26,27], there are several other exosome isolation methods [35]. Over time, there have been newer techniques developed for exosome isolation, as well as attempts to improve older, more traditional exosome isolation methods, including conventional ultracentrifugation [23,26,27,38]. For instance, when high-yields of pure exosomal preparations are needed, density gradient ultracentrifugation has been shown to be superior when compared to ultracentrifugation and other exosome isolation techniques [23,25,39,40,41]. Though all downstream applications do not require contaminant-free exosomes, it is noted that these preparations are highly desired when exosomes will later be used for exosomal RNA-seq and next generation sequencing [42,43,44]. However, density gradient ultracentrifugation is notorious for being difficult and tedious to perform [23,26,45,46]. Therefore, a potential alternative strategy to use to effectively isolate high-yields of pure (human urinary) exosomes that is simpler and more convenient than density gradient ultracentrifugation is ultracentrifugation followed by SEC column-purification.

There are two important items we want to emphasize about our study. One, we acknowledge that while our novel exosomal isolation approach which involves combining ultracentrifugation and SEC column-purification appears to work well when isolating exosomes from human urine, caution should be warranted when other types of biological fluid are being used to isolate exosomes. Indeed, it is advised that when SEC fractions are initially collected and originate from a biological fluid that is not human urine, then these fractions should be characterized similarly to what was performed in our respective study, before deciding upon which fractions are most appropriate to use for various downstream applications. While fractions 6 and 7 corresponded with which fractions were predicted to have robust, contaminant-free exosomes based on SEC column manufacturer instructions, it is certainly possible that the many other types of various SEC column-purification methods may result in other fractions containing large amounts of pure exosomes. Therefore, as an initial precaution, it is strongly suggested to analyze all collected fractions by characterization approaches outlined in this study, to better determine which fraction(s) contain large numbers of exosomes that are devoid of contamination.

In conclusion, our results indicate that human urinary exosomes isolated via ultracentrifugation followed by SEC column-purification provide fractions that contain similar numbers of exosomes when compared to human urinary exosomes that are isolated by ultracentrifugation only. However, human urinary exosomes that are isolated via ultracentrifugation alone are shown to have contamination present, while the exosome SEC column-purified fractions do not. Ultimately, for the human urinary exosomes to have any clinical value, a urinary exosomal biomarker or groups of biomarkers that aid in determining whether a patient is at risk of developing CKD, need to be identified. Indeed, if a specific microRNA or cluster of small RNA that is detected within intact human urinary exosomes are shown to increase CKD risk, then these biomarkers could be used clinically to augment other types of renal function tests [47,48]. Based on our results, which human urinary exosome isolation method should be utilized may ultimately depend upon whether exosomal preparations need to truly be contaminant-free or not for downstream purposes. If only a few small RNA of interest are attempting to be detected within intact human urinary exosomes, then conventional ultracentrifugation may be a simpler and straightforward method to use for exosome isolation, which can then be followed by an exosome-degradation assay to determine whether the small RNA of interest are actually protected within intact exosomes [22]. However, if researchers want to identify several potential diagnostic biomarkers for CKD (or other conditions) within human urinary exosomes through next generation sequencing, then opting to isolate human urinary exosomes by ultracentrifugation followed by SEC column-purification, would be a more appropriate approach compared to ultracentrifugation only, as the additional SEC step would likely prevent exosomal preparation contamination.

## Figures and Tables

**Table 1:** Participant Demographics.

Age	Sex	Race/Ethnicity	Weight	Height	BMI
13 y 0 m	F	Black/African American	85.5	1.627	32.30
11 y 6 m	M	Hispanic	60.1	1.478	27.51
8 y 3 m	M	Black/African American	94	1.334	52.82
7 y 6 m	M	Black/African American	40.4	1.306	23.69
15 y 1 m	F	Black/African American	106	1.645	39.17

## Data Availability

All data that is represented within this study is contained in the manuscript.

## Supplementary Materials

The following supporting information can be downloaded at: https://www.mdpi.com/article/10.3390/jpm12030340/s1, Figure S1: Unbiased assessment of human urinary particles isolated by ultracentrifugation alone versus ultracentrifugation coupled with SEC column-purification.; Figure S2: Unbiased assessment of transmission electron micrographs of a pooled human urinary exosomal preparation.
