# Supplementary material for: Comparison of Human Urinary Exosomes Isolated via Ultracentrifugation Alone versus Ultracentrifugation Followed by SEC Column-Purification
Source: J Pers Med. 2022 Feb 24;12(3):340. doi: 10.3390/jpm12030340 (PMC8950278; doi:10.3390/jpm12030340)
Supplement: Supplementary file 1 [file jpm-12-00340-s001.zip › jpm-1610416-supplementary.pdf]

Supplementary Figure Legends

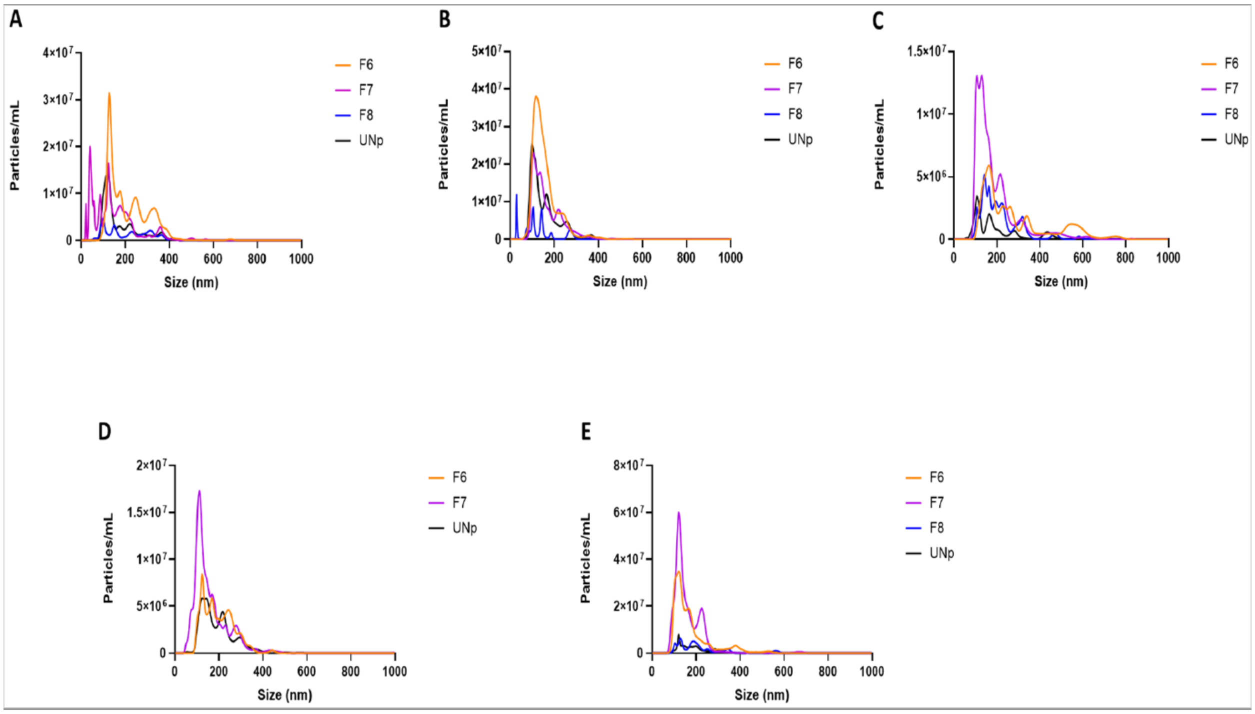

Supplementary Figure S1. Unbiased assessment of human urinary particles isolated by ultracentrifugation alone versus ultracentrifugation coupled with SEC column-purification. (**A–E**) Human urine was collected from participants listed in Table 1. Human urinary particle concentration and diameter size assessed using nanoparticle tracking analysis by NanoSight for the particles isolated via ultracentrifugation and remaining unpurified (UNp) or isolated by ultracentrifugation and then fractionated via SEC column-purification, with fraction 6 (F6), fraction 7 (F7), and fraction 8 (F8) being assessed. Supplementary Figure 1A–E data shown is identical to the data shown in Figure 1A–E, but supplementary data representation is unbiased due to a 1,000 nm size distribution being shown. (**D**) F8 was under the limit of detection.

A

B

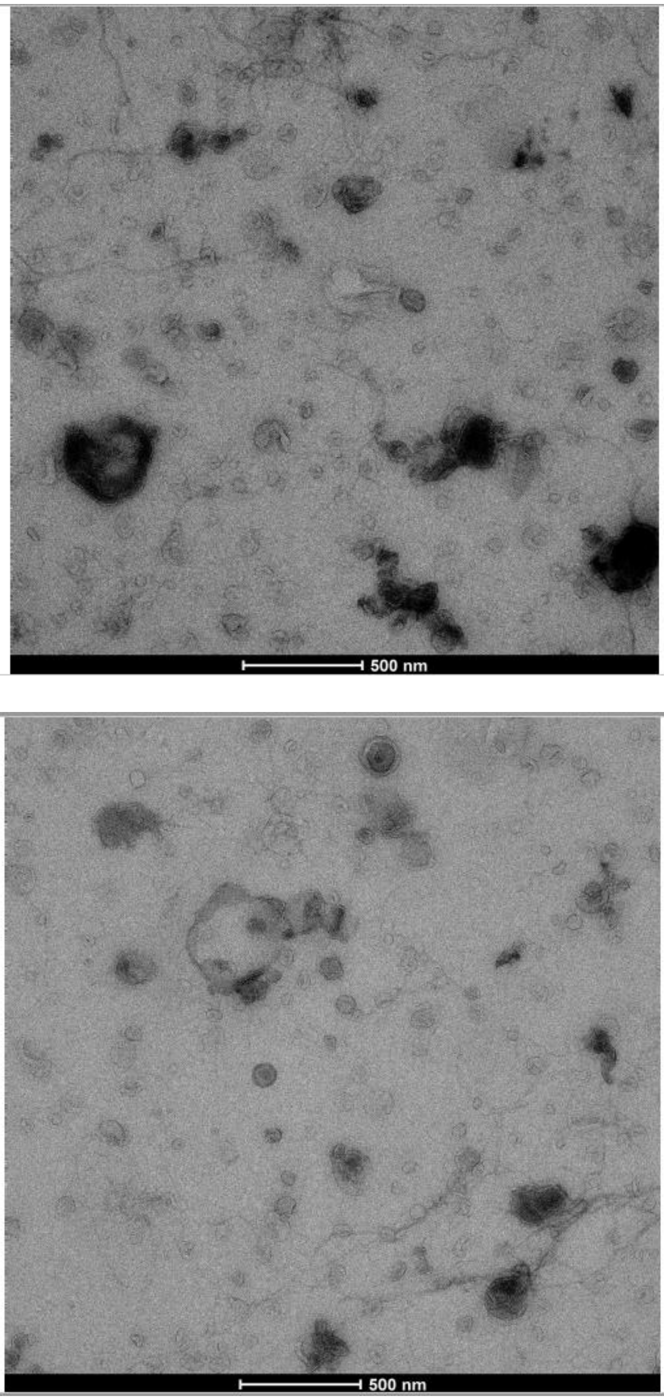

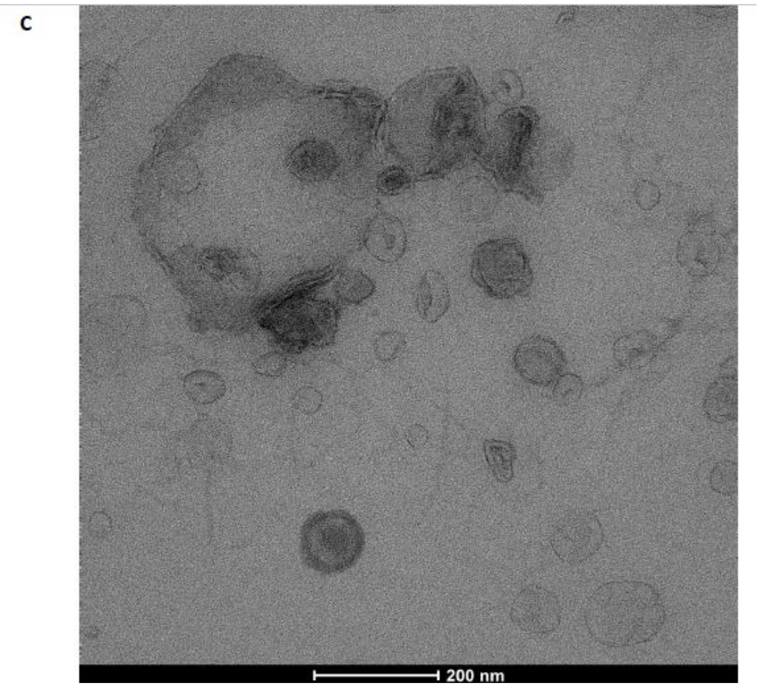

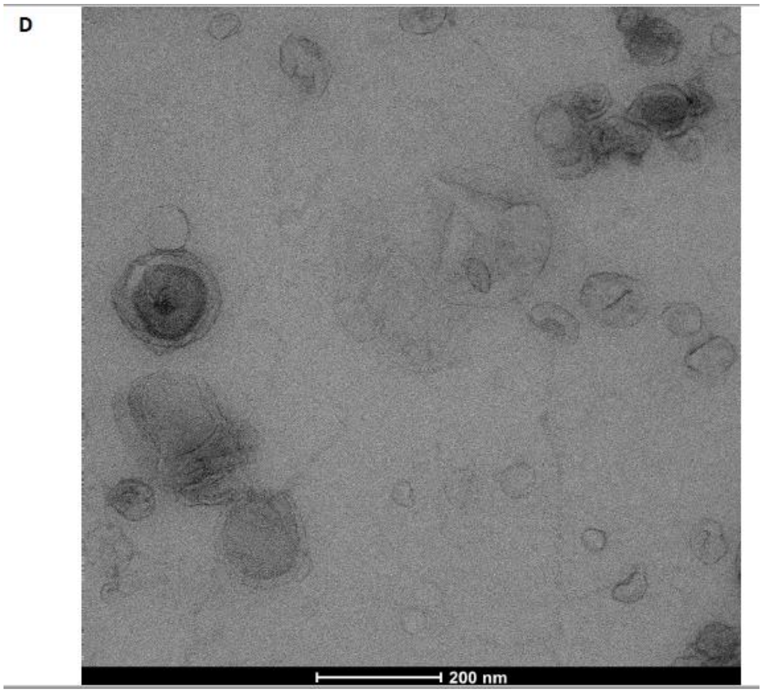

Supplementary Figure S2. Unbiased assessment of transmission electron micrographs of a pooled human urinary exosomal preparation. (**A–D**) Human urinary particles were isolated via ultracentrifugation coupled with SEC column-purification. All fraction 6 and fraction 7 samples became pooled, post-SEC column-purification and imaging was performed on the pooled

preparation using transmission electron microscopy. Supplementary Figure 3A–D images shown are the original, uncropped transmission electron micrographs, while Figure 3A,B exhibit cropped versions of the transmission electron micrographs.
